# Baseline beliefs about medication are associated with outcomes of antidepressants in inpatients with first-diagnosed depression under supervised therapeutic compliance

**DOI:** 10.18632/aging.203477

**Published:** 2021-09-02

**Authors:** Fan-Zhen Kong, Cai-Fang Ji, Xiang-Dong Du, Robert Logan, Hui-Ying Zhao, Guan-Hui Wu, Yan-Song Liu, Zhen Tang, Mei-E Niu

**Affiliations:** 1 Department of Psychology, Suzhou Guangji Hospital, The Affiliated Guangji Hospital of Soochow University, Suzhou, Jiangsu, China; 2 Biology, Eastern Nazarene College, Quincy, MA 02170, USA; 3 Department of Neurology, The Affiliated Suzhou Hospital of Nanjing Medical University, Suzhou Municipal Hospital, Gusu School, Nanjing Medical University, Suzhou, Jiangsu, China; 4 Department of Nursing, The First Affiliated Hospital of Soochow University, Suzhou, Jiangsu, China

**Keywords:** first-diagnosed depression, medication beliefs, BMQ-S, antidepressants, therapeutic compliance

## Abstract

The aim of the present study was to explore the effect of baseline beliefs about medication on therapeutic outcomes of antidepressants in inpatients with first-diagnosed depression under supervised therapeutic compliance. Ninety-seven inpatients with first-diagnosed depression were included to collect their baseline demographic data to evaluate the Hamilton depression rating scale (HAMD) scores and the beliefs about medicine questionnaire-specific (BMQ-S) scores at baseline and the end of the eight-week treatment. Additionally, we explored the relationship between inpatients’ medication beliefs and therapeutic effect of antidepressants. The inpatients were divided into remitted depression and unremitted depression groups according to outcomes at the end of the eight-week treatment. There was no significant difference in the baseline HAMD between the two groups (*P* > 0.050). The scores on the BMQ-S of the unremitted group were significantly lower than those of the remitted group (*P* < 0.001). The HAMD scores were significantly reduced in both groups after the eight-week treatment (*P* < 0.001). There was no significant difference in the BMQ-S scores before and after the treatment (*P* > 0.050). The medication beliefs of the unremitted inpatients after the treatment were still lower than those of the remitted inpatients (*P* < 0.001). Logistic-regression analysis showed that low BMQ-S scores at the baseline were an independent risk factor for antidepressant efficacy. Beliefs about medication at baseline may be correlated with the therapeutic efficacy in inpatients with first-diagnosed depression under supervised therapeutic compliance.

## INTRODUCTION

Depression is one of the most common, harmful, and burdensome mental illnesses. Approximately 5–12% of males and 9–26% of females will experience at least one depressive episode in their lifetime, and approximately 50% of depressed patients will experience a second depressive episode [1–3]. Patients with two or more episodes have a 70–90% chance of recurrence of depression concomitant with poor prognosis [4, 5]. Drug treatment is the most important therapeutic approach for depression [6, 7]. However, many patients do not respond well to the medications or do not tolerate the side effects. Approximately half of the patients who initially receive monotherapy show an efficacious response, and only approximately one-third of the patients obtain long-lasting relief from depression [8]. Ten to twenty percent of depressed patients who do not get relief progress to treatment-resistant depression [9, 10].

Poor medication adherence is one of the factors influencing the progression to treat-resistant depression [11–13]. Compliance with medications depends at least in part on the patient’s beliefs about antidepressant medication [14–16]. Beliefs about medication refer to the patient’s perceived necessity and their concerns about taking the medication. The scores on an evaluation represent the difference between a patient’s necessity beliefs and concern beliefs about using the medication and reflects the patient’s own judgment on the relationship between the pros and cons of the drug treatment [17]. Necessity beliefs refer the patient’s perception that using medication is beneficial to control disease, relieve symptoms, prevent recurrence, and improve prognosis and the quality of life. Concern beliefs refer to the patient’s concerns about drug efficacy, side effects, drug dependence, and drug addiction. The effects of medication beliefs on therapeutic compliance have been widely recognized but the direct effects on therapeutic outcome are not well established [18–21].

Standard drug treatment for patients with first-diagnosed depression is the main therapeutic measure for reducing the recurrence rate of depression and preventing progression to treatment-resistant depression [22]. Effects of medication beliefs on the direct effect of therapeutic outcomes excluding the compliance influence for inpatients with first-diagnosed depression have not been reported previously. Hence, the present study explored the characteristics of medication beliefs in inpatients with first-diagnosed depression and the direct effects on the therapeutic outcomes of antidepressants through monitoring the medication by nurse to provide reference for treatment decision.

## MATERIALS AND METHODS

### Research subjects

Patients with first-diagnosed depression were individually recruited by experienced psychiatrists from outpatient and inpatient department of Psychology, Suzhou Psychiatric Hospital in Jiangsu Province from January 2018 to December 2019. The inclusion criteria for patients were as follows: (1) met the diagnostic criteria for depression listed in the Diagnostic and Statistical Manual of Mental Disorders, Fifth Edition (DSM-5) published by the American Psychiatric Association; (2) scores > 17 on the 17-item Hamilton depression rating scale (HAMD-17); (3) ages from 18 to 60 years old; (4) patients were not treated for antidepressants before hospitalization and regularly taking medication under the supervision of the nurse for eight weeks. (5) signed the informed consent. The exclusion criteria of this study were as follows for the candidate patients: (1) depression caused by other diseases, such as bipolar depression; (2) depression accompanied by severe organic brain disorder; (3) severe physical illness; (4) other psychiatric disorders; (5) high risk for suicide (≥ 3 suicide score on HAMD-17); or (6) pregnancy or lactation. The 17-item HAMD scores and beliefs about medicine questionnaire-specific (BMQ-S) scores at the baseline and the end of the eight-week treatment were examined and divided into the remitted group (Remitted) and Non-remitted group(Non-Remitted) groups according to the therapeutic outcomes. This study included a total of 97 patients with first-diagnosed depression, including 39 males and 58 females, with their ages ranging from 18–60 years old (mean age: 39.00 ± 12.29 years). The study was approved by the Ethics Committees of the Suzhou Psychiatric Hospital, The Affiliated Guangji Hospital of Soochow University, and all participants signed an informed consent form. All study procedures were in accordance with the Declaration of Helsinki.

### Evaluation tools

#### Self-rated questionnaires

Self-rated survey questionnaires were used to collect the basic clinical data of the participants, such as age, sex, disease course, years of education, marital status, economic condition and previous medication. Married or cohabiting for more than 6 months means in marital status, otherwise unmarried. Economic status poverty refers to the family per capita annual income of less than 3,000 yuan (RMB), or higher income. Previous medication history was divided into: 0, no medication; 1, sleeping pills; 2, other drugs (drugs for other non-mental diseases); 3, 1 and 2.

#### HAMD-17

The HAMD-17 was used to evaluate the severity of depression. Most of the items in the scale were evaluated using a five-point scale (0–4 scores), and some individual items were evaluated using a three-point scale (0–2 scores). The total score was the sum of all items in the scale. Patients were divided into two groups based on the total score of the HAMD-17, including the severe-depression group (with total scores > 24) and the mild-to-moderate depression group (with total scores > 17 but ≤ 24).

#### BMQ-S

The BMQ-S, established by Horne in 1999, is mainly used to assess the beliefs about medication [17] and consists of two dimensions: (1) necessity beliefs and (2) the concern beliefs about medication. Each dimension contained five items, and each item was evaluated using a five-point scale (1–5 scores). The total score obtained in each dimension was divided by five, with the score ranging from 1 to 5 (the higher the score, the stronger the relevant beliefs), and the final result was calculated according to the equation as follows: score of beliefs about medication equals the score of necessity beliefs minus the score of perceived concern beliefs.

### Statistical analysis

SPSS24.0 software (IBM SPSS Inc., Chicago, IL) was used for statistical analysis in this study. The normally distributed data were analyzed by *t*-tests or analyses of variance and are presented as mean ± standard deviation $\left(\bar{x}\;\pm \text{ s}\right)$. The counted data were analyzed by chi-square tests and are presented as the numbers of cases (percentages). The HAMD-17 and the BMQ-S scores between the two groups before and after the treatment were compared using repeated-measures analysis of variance. Logistic-regression analysis was used to analyze the risk factors associated with non-remitted and control the confounding factors. Sensitivity analysis by varying the number of confounders showed that the results of this study were stable. *P* < 0.05 was considered as a statistically significant difference.

### Ethics approval and consent to participate

All participants gave written informed consent and ethical approval was obtained from the Ethics Committee of Suzhou Guangji Hospital (2017-036).

### Availability of data and materials

The dataset used and analysed during the current study are available from the corresponding author on reasonable request.

## RESULTS

### Data comparison between the patients with non-remitted and remitted at baseline

According to the HAMD score at the end of the eight-week treatment, there were 76 remitted cases and 21 non-remitted cases among the 97 patients with depression. No significant differences in age, sex, disease course, years of education, marital status, economic conditions, previous medication or HAMD scores were found between the two groups of patients (*P* > 0.05). The patients with non-remitted had significantly lower BMQ-S scores than those of the patients with remitted (*P* < 0.001, Table 1).

**Table 1:** Data comparison between the patients with Non-remitted and Remitted at baseline.

	**Remitted (*n* = 76)**	**Non-Remitted (*n* = 21)**	**Test value**	***P* value**
Age	39.64 ± 12.15	38.52 ± 13.07	0.368	0.714
Sex (female)	43 (56.6%)	15 (71.4%)	1.509	0.219
Duration (months)	7.80 ± 4.48	8.67 ± 4.05	−0.798	0.427
Education (years)	12.00 ± 3.63	10.76 ± 3.56	1.404	0.170
Marital status (married)	51 (67.1%)	16 (76.2%)	0.636	0.425
Income (good)	52 (68.4%)	14 (66.7%)	0.023	0.879
Medication				
None	47 (61.8%)	12 (57.1%)	0.135	0.713
Sleep aids	10 (13.2%)	5 (23.8%)		
Others	9 (11.8%)	3 (14.3%)		
Sleep aids + Others	10 (13.2%)	1 (4.8%)		
Baseline HAMD	25.34 ± 5.11	23.43 ± 5.12	1.559	0.122
Baseline BMQ-S	1.05 ± 1.24	−1.17 ± 2.10	4.828	0.000

### Comparison of HAMD and BMQ-S scores in the two groups of patients between before and after antidepressant treatment

Repeated-measures analysis of variance of the HAMD scores showed significant differences in the main effect between groups (F = 4.958, *P* = 0.028), the main effect of time (F = 808.859, *P* < 0.001), and mutual effect between groups and time (F = 36.982, *P* < 0.001). Pairwise comparisons showed that there was no significant difference in HAMD scores between groups before the treatment (t = 1.559, *P* = 0.122) but that there was a significant difference in HAMD scores between groups after the treatment (t = −6.295, *P* < 0.001). A significant difference in the HAMD score was found within each group before and after antidepressant treatment (t = 31.548, 9.701; both *P* < 0.001; Table 2).

**Table 2:** Comparison of HAMD and BMQ-S scores between the two groups of patients before and after antidepressant treatment.

	**HAMD**		**BMQ-S**	
	**Pre-treatment**	**Post-treatment**	**Pre-treatment**	**Post-treatment**
Remitted (*n* = 76)	25.34 ± 5.11	4.66 ± 2.38^#^	1.05 ± 1.24	1.21 ± 1.85
Non-Remitted (*n* = 21)	23.43 ± 5.12	10.04 ± 3.88^*#^	−1.17 ± 2.10^*^	−0.78 ± 2.13^*^

Compared to Remitted, ^*^P < 0.001; Compared to Pre-treatment, ^#^*P* < 0.001.

Repeated-measure analysis of variance of the BMQ-S scores showed a significant difference in the main effect between groups (F = 36.969, *P* < 0.001); however, there were no significant differences in the main effect of time (F = 1.629, *P* = 0.205) or in the mutual effect between groups and time (F = 0.279, *P* = 0.598). Pairwise comparisons showed significant differences in BMQ-S scores between groups before and after antidepressant treatment (t = 4.828, 4.359; both *P* < 0.001). No significant difference in BMQ-S scores was found within each group before or after antidepressant treatment (t = −0.725, −0.517; *P* = 0.470, 0.608; Table 2).

### Analysis of independent risk factors for non-remitted patients

The outcomes of the eight-weeks antidepressant treatment (non-remitted and remitted) were considered as dependent variables, and the age, sex, disease course, years of education, marital status, economic conditions, previous medication, HAMD scores and BMQ-S scores at baseline were considered as independent variables. The variable screening was performed by the Enter method. Multivariable logistic regression analysis showed that the BMQ-S scores at baseline were independently and negatively correlated with the treatment outcomes (OR: 0.386, *P* < 0.001, 95% CI: 0.257–0.579; Table 3).

**Table 3:** Analysis of independent related factors of Non-Remitted depressed patients.

	**B**	**SE**	**Wald**	**P**	**OR**	**95%CI**
Age	0.029	0.034	0.761	0.383	1.030	0.964~1.100
Sex (female)	0.148	0.761	0.038	0.846	1.159	0.261~5.149
Duration (months)	−0.005	0.102	0.002	0.962	0.995	0.814~1.216
Education (years)	−0.184	0.111	2.717	0.099	0.832	0.669~1.035
Marital status (married)	−0.270	0.828	0.107	0.744	0.763	0.151~3.867
Income (good)	−0.315	0.896	0.124	0.725	0.730	0.126~4.221
Medication usage						
None	−	−	4.310	0.230	−	−
Sleep aids	0.935	1.031	0.821	0.365	2.547	0.337~19.229
Others	0.938	1.047	0.802	0.370	2.554	0.328~19.878
Sleep aids + Others	−2.306	1.453	2.518	0.113	0.100	0.006~1.720
Baseline HAMD	0.139	0.076	3.315	0.069	1.149	0.989~1.334
Baseline BMQ-S	−0.225	0.055	17.110	0.000	0.798	0.717~0.888
Constant	−3.882	2.843	1.864	0.172	0.021	−

## DISCUSSION

In patients with first-diagnosed depression, the initial beliefs about medication were the only contributing factor to the short-term therapeutic efficacy of antidepressants. This result indicated that non-remitted patients had stronger concern beliefs than necessity beliefs prior to treatment. Antidepressants alleviated the depressive symptoms of the patients but did not change the patients’ beliefs about medication.

Depressed patients with stronger medication beliefs at baseline were more likely to obtain symptomatic relief after antidepressant treatment but non-remitted patients with weaker medication beliefs at baseline less likely to be relieved, suggesting that the therapeutic outcomes of depressed patients were highly correlated with their initial beliefs about medication. Depressed patients with suicidal attempt have weaker baseline necessity beliefs, and suicidal patients reporting hopelessness during follow-up have relatively strong concern beliefs [23]. Weak necessity beliefs and strong concern beliefs about medication are closely related to poor adherence while therapeutic compliance is an important intermediary factor between the medication belief and treatment efficacy [24–26]. The medicine administration in this cohort is supervised by designated nurse, who guarantees the timeliness and accuracy of the patient taking the medicine, eliminates the influence of compliance differences outside of the hospital, directly investigates the relationship of the baseline medicine belief and the treatment effect, and ensures the reliability of the result. This result confirmed the findings of previous studies that medication beliefs affect the antidepressant efficacy. Providing patients of poor medication beliefs targeted psychological health education with correct perception on antidepressants may improve their compliance and prognosis [16, 27–31]. This highlights the importance of screening for medication beliefs.

Our study showed that the severity of depression in patients was improved after antidepressant treatment, but patients’ beliefs about medication were not changed following treatment. Previous studies have rarely reported the effects of antidepressant treatment on patients’ medication beliefs, and the factors affecting beliefs about medication are poorly understood. Lesén et al. [23] have shown that suicidal patients with severe anxiety symptoms have strong necessity beliefs about medication, while patients with extroverted personalities had insufficient awareness of the necessity of taking medication, suggesting that personality traits may be an important factor affecting beliefs about medication, whereas the changes in the severity of depression before and after antidepressant treatment have no significant effect on beliefs about medication. Lemay et al. [26] have shown that weak medication beliefs are associated with unmarried status, ethnicity, lower education levels, and relatively severe illness. In the present study, no significant differences were found in terms of race, education level, marital status, economic condition, previous medication or disease severity at baseline between the two groups of patients, but the beliefs about medication were different between the two groups of patients. This difference may be due to other factors, such as personality traits, whether they have insurance to pay for the medication, perception of the medication, or stigma [23, 26, 32].

The present study showed that beliefs about medication were the only influencing factor affecting the short-term therapeutic outcomes in patients with first-diagnosed depression. No similar reports were published in previous depression-related studies. Zayed et al. [33] have shown that beliefs about medication are the only predictor of therapeutic compliance in patients with Behcet’s disease, which is similar to the results of our current findings for depressed patients. There are differences in the impact of necessity beliefs and concern beliefs on therapeutic compliance in different studies. Most studies have shown that necessity beliefs were positively correlated with higher medication compliance while concern beliefs were positively correlated with poor compliance of patients [16, 34–36]. High medication compliance is correlated with the improvement of depressive symptoms in some patients. Lemay et al. [26] have shown that negative medication beliefs, young age, and severe illness are influencing factors of poor medication compliance in patients with chronic diseases. The previous studies examined the indirect efficacy of medication beliefs through the intermediary effects of the compliance on the prognosis of the disease, without examining the direct potential value of the belief on the therapeutic outcome. In the present study, patients with post-treatment remission had stronger necessity beliefs than concern beliefs at baseline; in contrast, non-remitted patients had weaker necessity beliefs than concern beliefs at baseline, suggesting that the total score of BMQ-S may be a decisive prognostic factor on therapeutic effect. Under the supervision of nurses, the patient's medication compliance was close to 100% which guarantees that this cohort eliminates the intermediary influence of compliance differences between medication beliefs and the therapeutic outcome. Based on above work, the prediction of medication beliefs on the treatment outcome may have an additional mechanism independent of compliance, such as whether the scale itself has the effect of predicting the potential anxiety of the patients, which deserves further study. The potential value of the scale may also be one of the reasons why this study found that medication belief is the only predictor of the treatment outcomes.

The present study has certain limitations. Firstly, this is a retrospective in hospital investigation. Selection bias may result from outpatients’ intention to be hospitalized. The results need to be interpreted with caution. Secondly, this study didn’t analyze the correlation between beliefs of necessity or concern and the treatment outcome, and therefore could not determine the specific role of each factor on the prognosis. Previous papers have examined the effects of the two factors on compliance and prognosis respectively, and this study has examined the integrated impact of patients’ medication belief on treatment outcome. Therefore, the results of this study have important complementary value to previous studies. Thirdly, the study didn’t perform hierarchical analysis of the patient’s initial antidepressants, and differences in the effects of variant antidepressants may affect the prognosis of patients. Fourthly, the mechanism by which medication beliefs influence short-term prognosis of depression independently of compliance remains to be clarified. These unmet clinical needs need to be addressed in future large-scale clinical studies. Nevertheless, the medication regimen of this study follows the treatment guideline, the 8-week hospitalization period not only ensures the patient’s medication compliance, but also provides a reliable medical record for judging the effect. The clear results of this study deepen the understanding of the influence of medication belief on the prognosis of patients with depression.

This study confirms the significant effect of medication belief on the outcome of antidepressant treatment. For the first time, this study raises the possibility that the effect of medication belief on the antidepressant outcome may have additional mechanism independent of therapeutic compliance. The results of this study warrant further studies on excavating the potential value of the scale for developing new strategies to treat depression.
